# Hardware Failure Prediction on Imbalanced Times Series Data: Generation of Artificial Data Using Gaussian Process and Applying LSTMFCN to Predict Broken Hardware

**DOI:** 10.1007/s10278-020-00411-4

**Published:** 2021-01-06

**Authors:** Nadine Rücker, Lea Pflüger, Andreas Maier

**Affiliations:** Pattern Recognition Lab, FAU Erlangen-Nürnberg, Erlangen, Germany

**Keywords:** Time series classification, Gaussian process regression, Machine learning, Hardware failure prediction

## Abstract

Magnetic resonance imaging (MRI) systems and their continuous, failure-free operation is crucial for high-quality diagnostics and seamless workflows. One important hardware component is coils as they detect the magnetic signal. Before every MRI scan, several image features are captured which represent the used coil’s condition. These image features recorded over time are used to train machine learning models for classification of coils into normal and broken coils for faster and easier maintenance. The state-of-the-art techniques for classification of time series involve different kinds of neural networks. We leveraged sequential data and trained three models, long short-term memory (LSTM), fully convolutional network (FCN), and the combination of those called LSTMFCN as reported by Karim et al. (*IEEE access* 6:1662–1669, 2017). We found LSTMFCN to combine the benefits of LSTM and FCN. Thus, we achieved the highest F1-score of 87.45% and the highest accuracy of 99.35% using LSTMFCN. Furthermore, we tackled the high data imbalance of only 2.1% data collected from broken coils by training a Gaussian process (GP) regressor and adding predicted sequences as artificial samples to our broken labelled data. Adding 40 synthetic samples increased the classification results of LSTMFCN to an F1-score of 92.30% and accuracy of 99.83%. Thus, MRI head/neck coils can be classified normal or broken by training a LSTMFCN on image features, successfully. Augmenting the data using GP-generated samples can improve the performance even further.

## Introduction

Even the best hardware can fail. Our goal is to predict failures as early as possible. The majority of systems nowadays log measurements during operation. These measurements and their deviations from the norm contain valuable information. This enables us to detect malfunctioning hardware components. In particular, in medical imaging devices, such as magnetic resonance imaging (MRI), this is of high interest. In MRI, coils consist of conductive wires and detect the MR signal. The resulting image highly depends on the coil’s condition. In this work, we use key performance indicators (KPIs) which are measured using head/neck coils right before the actual medical imaging procedure starts.

The classical supervised learning problem is to predict the correct class of new objects after training on objects with given classes [2]. Models like linear probabilistic models, neural networks, kernel methods, and graphical models can be applied [3]. Evolving from single instances, a lot of data is also collected over time, e.g. weather readings. This enlarges the dimension of input data and requires slightly different methods.

In literature, time series classification is widely discussed. Deep learning is found to be very successful [4]. A recurrent neural network (RNN) can take sequences as input or output, or even both.

One special setup of recurrent neural network has already been introduced in 1997 called long short-term memory (LSTM) [5] and experiences great success in various applications, e.g. language modelling [6] or human activity recognition [7]. Furthermore, fully convolutional neural networks (FCNs) are introduced for the classification of univariate time series [8]. Taking these concepts even further, FCN is augmented with LSTM sub-modules and outperforms the performance of regular FCNs [1].

Independent of the chosen algorithm, a common problem in machine learning (ML) classification is imbalanced classes. In order to overcome the disproportionate ratio of observations in each class, possible options are sampling techniques, modification of classification methods, or generating synthetic samples [9]. For example, an infinitely imbalanced logistic regression is applied to an imbalanced data set in order to improve mine classification [10]. As another example, samples are generated with variational Bayesian specific for image classification [11].

Previous research on MRI failures has rather focussed on artefact detection and classification [12]. Several ML algorithms are applied to image features of MRI systems for hardware failure prediction [13, 14]. However, sequential data was not used, and thus, the likely interdependence of features over time was not considered. Other authors predict the time until hardware components failed [15]. Furthermore, in previous research, we showed that LSTM outperformed other algorithms like FCN or residual neural network when applied to time series data, however, struggled with data imbalance [16]. LSTMs are also applied on clinical time series data in order to predict the diagnosis [17].

## Materials and Methods

We set up a machine learning pipeline in order to identify suspicious or already broken MRI coils. For that, we leverage a sequence of input data and desire a label hinting to broken or normal as output. Therefore, we train and test a LSTM, FCN, and LSTMFCN on image features recorded by head/neck coils. First, we describe and present the underlying data. Furthermore, we explain the needed preprocessing steps. Afterwards, the applied models are discussed.

### Data

**Fig. 1:**
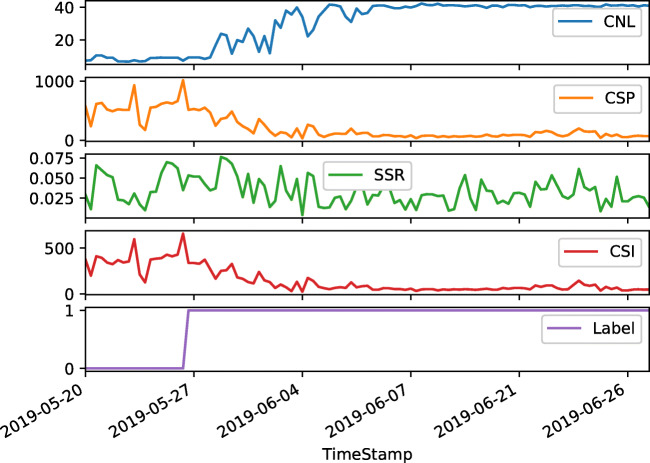
Temporal progression of the four numerical KPIs and the corresponding label of one exemplary coil. The label switch from 0 to 1 denotes the transition from normal to broken

We apply our methods onto sequential data acquired by 68 Siemens MRI scanners each using one or several different 20-channel head/neck coils. Before every MRI examination, coil adjustment measurements are performed which deliver KPIs representative for image features. We collect four numerical, one-dimensional features per coil channel. They depict the channel signal noise level (CNL), channel signal to noise ratio (CSP), channel signal to signal ratio (SSR), and the channel signal to noise ratio at isocenter (CSI). Data is acquired over the period of six months from 57 coils without failures and from 11 coils which break over the course of our recording. This yields in 361,558 samples which contain 2.1% samples from broken coils. The features and their record over time do not allow any reconstruction of medical or patient-specific features. Thus, we work on fully anonymous, non-clinical data. Figure 1 shows the four features over the course of more than 1 month for one exemplary system. The fifth chart displays the respective label jumping from normal (represented by the value 0) to broken (indicated with the value 1). Please note that the time axis only holds timestamps where measurements are available; thus, it does not reflect a continuous time scale.

### Data Preprocessing

**Fig. 2:**
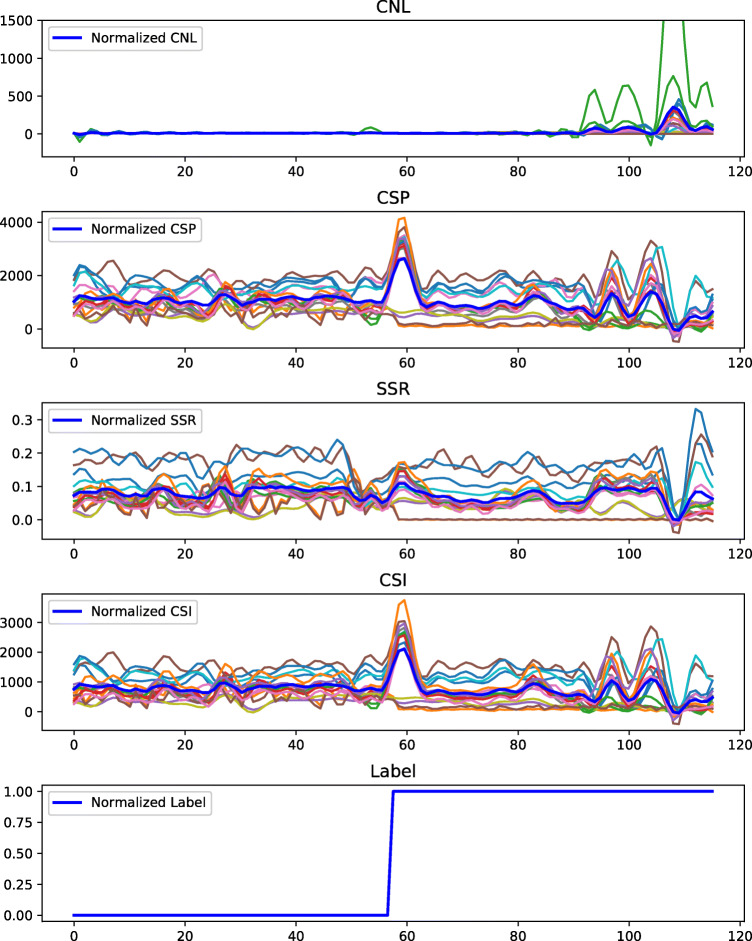
Example of one artificially generated sequence drawn as a thick, dark line next to original sequences. The synthetic sequence was predicted using a Gaussian process regressor for the respective KPIs, individually

First, we normalize the measurements per feature by subtracting the mean and dividing by the standard deviation, respectively. The time series of measurements exhibit different lengths as the underlying MRI systems were used in various frequencies. A total of 40 samples represent 1 day of measurements on average. Thus, we split the individual vectors into chunks of the unified length of 40 measurements. These chunks are created by applying sliding windows resulting in 31,834 normalized training sequences. As soon as a training sequence contains one measurement from a broken coil, the sequence is labelled broken. In the next step, we address the problem of high data imbalance, as only 680 (2.1%) normalized sequences were measured from broken coil elements. In order to increase the number of samples of broken coils, we applied a Gaussian process (GP) regressor [18] and modelled the relationship between label and measured KPIs. The Gaussian process is entirely defined by its mean and covariance function. In the course of this paper, a radial basis function (RBF) is used as the covariance function. Thus, we fit a Gaussian process to our data using maximum likelihood estimation of the parameters. For that, we collect available sequences of breaking coils and overlay the time stamp of label switch. Then we predict samples for each feature individually from its learnt regressor and estimate new, broken sequences. We visualize the original sequences next to one exemplary artificially created sequence drawn as thick, dark line in Fig. 2. The visible spikes are recorded when images denote higher noise levels than normal. These can be caused by various circumstances, e.g. electrical sparks, presence of signal disturbing equipment, electronic interferences in the receiver circuits, or hardware failure. We record spikes not being produced by hardware failure as well as breaking coils without spikes. Thus, we rely on the change of the signal over time to detect breakage, reliably.

### Machine Learning Model

We use machine learning to classify MRI hardware as normal or defective. Our models are trained on image features recorded by MRI coils over time. As the amount of training data is limited, we apply leave-several-coils-out cross-validation with fivefolds. One fold contains samples from 68 coils where of 13 or 14 coils are left out for testing. The distribution per fold is given in Table 1. Thus, sequences from distinct coils were used for training and testing. We aim to detect failing hardware as soon as the first measurement is performed using one single broken coil element. This is implemented using sliding windows of the given sequences. Thus, broken hardware shall be detected already before the actual patient scan would start.

**Table 1:** Distribution of normal and broken samples in test set of each fold

Fold	0	1	2	3	4	Sum
Normal	4437	7020	6917	5246	7534	31154
Broken	397	14	87	50	132	680
Sum	4834	7034	7004	5296	7666	31834

Every fold contains training samples from 68 coils, leaving 13 or 14 coils out for testing

As we leverage sequential image features collected during runtime of the MRI systems and consequently of the used coils to predict the coil’s condition (broken or normal), we employ time series classification methods. One state-of-the-art technique to classify time series data are fully convolutional neural networks (FCNs). In our earlier research [16], we found LSTM and FCN to outperform time convolutional neural networks and residual networks. Thus, we use the combination of both, LSTM and FCN (LSTMFCN), to leverage the benefits of both and achieve a most accurate classification as proposed in [1]. In the following, we present the model details of LSTMFCN and the two individual models for comparison. We tuned and determined all hyperparameters per model individually, using the F1-score as the decisive metric.

#### Long Short-term Memory

For comparison, we implemented a LSTM network according to our previous reseach [16]. The model contains two convolutional layers without padding operations. We apply local average pooling and a dropout operation to prevent overfitting after each convolutional layer. This is followed by two LSTM layers with 32 units. Finally, we employ a dense layer with sigmoid activation function to calculate the result.

#### Fully Convolutional Network

Furthermore, a fully convolutional neural network (FCN) is built using three convolutional blocks as suggested in [8]. Each block contains one convolution, batch normalization, and ReLu activation layer. This is followed by global average pooling and a dense layer using softmax activation. We did not use any pooling to prevent overfitting nor a regularizer.

#### Long Short-term Memory Fully Convolutional Network

**Fig. 3:**
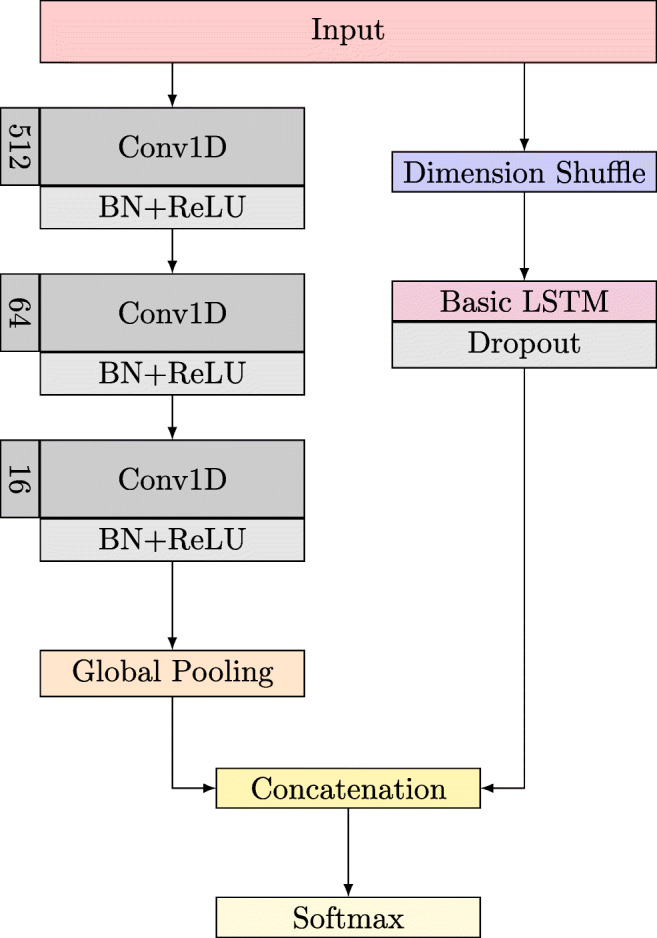
The LSTMFCN model architecture with respective filter sizes after hyperparameter tuning. It concatenates a standard FCN (left part) and LSTM (right part) and finishes with a softmax layer

Finally, a FCN is combined with the benefits of a LSTM. Therein, the FCN is augmented by LSTM as illustrated in Fig. 3. The FCN part contains three temporal convolutional blocks with filter sizes of 512, 64, and 16, respectively. Each of those blocks is followed by batch normalization and ReLU activation function. After the third convolution block, a global average pooling layer is induced. This is concatenated with the LSTM part which consists of a dimension shuffle first, followed by the main LSTM block and a Dropout layer. After concatenation, finally, the result is calculated using a softmax layer.

## Results

**Fig. 4:**
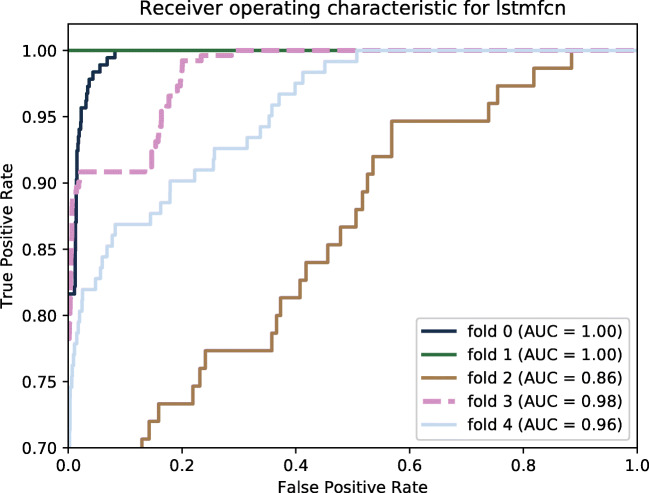
ROC curve for LSTMFCN applied onto the original data set given per fold

**Fig. 5:**
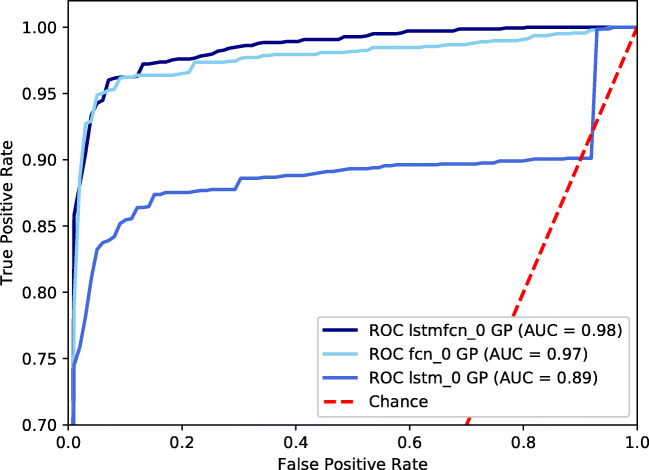
ROC curves of the three tested models after training on original data set without adding synthetic data

First, we compare the models amongst each other on the original, imbalanced data set. Table 2 shows the resulting performance measures for LSTM, FCN, and LSTMFCN next to each other as well as the confusion matrix. Those numbers present the averages over all fivefolds. TN holds values for the true negative rate, FP covers false positive rate, and FN and TP denote false negative and true positive rates, respectively. Furthermore, we provide the receiver operating characteristic (ROC) curve and the area under the ROC curve (AUC) of LSTMFCN for the different folds (see Fig. 4). In order to compare the different models, Fig. 5 holds ROC curves of LSTMFCN, FCN, and LSTM applied onto data without augmentation.

**Table 2:** Average prediction performance measures for our models applied to data set without augmentation

%	Accuracy	Precision	Recall	F1	TN	FP	FN	TP
LSTM	98.19	96.62	56.44	64.50	99.97	0.03	43.56	56.44
FCN	99.27	96.80	77.93	84.51	99.70	0.30	22.07	77.93
LSTMFCN	99.35	98.23	80.70	87.45	99.86	0.14	19.30	80.70

**Fig. 6:**
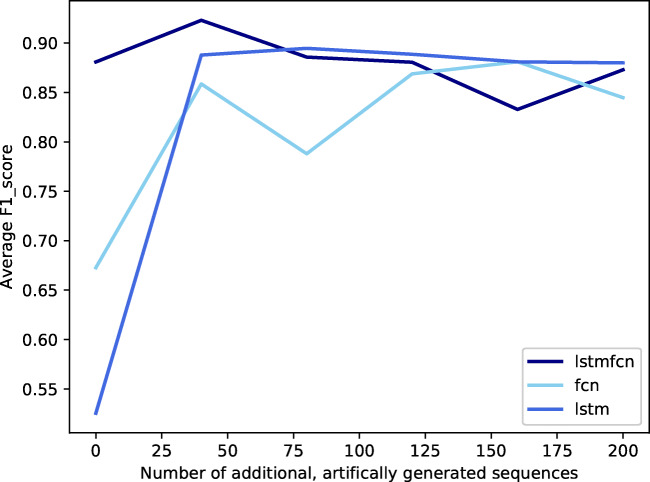
F1-scores for all three models after training on different amounts of added artificial, GP-generated sequences

**Fig. 7:**
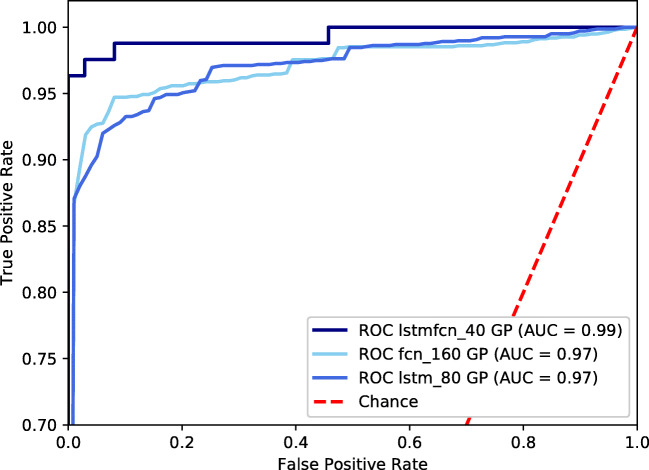
ROC curves of the three tested models. The underlying training data set includes 40 Gaussian Process–generated sequences

We continued experiments regarding the imbalance of our data and explored the behaviour of our three models. Thus, we added synthetic data by predicting sequences based on the GP regression model. We used different amounts of artificial data, ranging from 0 to 200 sequences. The resulting F1-scores of the three considered models are provided in Fig. 6. We found the highest F1-score of 92.3% for LSTMFCN using 40 GP data. The FCN achieved its highest F1-score of 88.1% after adding 160 synthetic sequences. Moreover, we yield an F1-score of 89.5% when applying LSTM onto our data set containing 80 additional sequences. The respective ROC curves of the models using their optimal number of added, artificial data are presented in Fig. 7.

As we found LSTMFCN reaching the highest F1-score of 92.3% by adding 40 synthetic sequences (equal to 2.3% data from broken coils), the detailed performance measures are provided in Table 3. The first row holds performance results of the LSTMFCN without artificial data for comparison.

**Table 3:** Average prediction performance measures for LSTMFCN applied onto the original data set as well as after adding 40 and 200 synthetic sequences

Broken								
samples	Accuracy	Precision	Recall	F1	TN	FP	FN	TP
2.1% (0)	99.35	98.23	80.70	87.45	99.86	0.14	19.30	80.70
2.3% (40)	99.83	100.00	85.70	92.30	100.00	0.00	14.30	85.70
2.9% (200)	98.67	92.46	89.53	90.87	99.29	0.71	10.47	89.53

## Discussion

We performed several experiments to find the best model for our problem of broken coil classification. We compared three different models, LSTM, FCN, and the combination of both, called LSTMFCN. The performance results support the strategy of combining the benefits of LSTM and FCN in LSTMFCN. Thus, Table 2 shows that LSTMFCN outperforms LSTM and FCN in all presented performance measures, accuracy, precision, recall, and F1-score. As we deal with highly imbalanced data, F1-score is the most meaningful measure and reaches 87.45%. However, FCN results are very close to LSTMFCN. The confusion matrix gives further insights into LSTM’s classification issues of missing out a lot of broken coils. Using LSTM, on average almost every second broken coil is classified falsely as represented by a false negative rate of under 44%. The FCN achieves better performance than LSTM, however does not reach LSTMFCN’s average performance, neither. We see also here only a true positive rate of 77.93% being lower than 80.70% reached using LSTMFCN. If we have a closer look at the ROC curves (Fig. 5), FCN and LSTMFCN curves are very close, whereas LSTM falls behind. We denote the high ability of classifying correctly using FCN and LSTMFCN to the nature of the three subsequent convolution layers. Paying attention to the individual folds and their performance presented in Fig. 4, fold 2 stands out with the lowest performance. That fold contains the largest proportion of coils which brake at a late point in time of our recordings. This makes the amount of sequences from broken coils smaller than those in the other folds.

Consequently, we generated synthetic samples to increase the number of broken coil KPIs. As presented in Fig. 6, adding artificial data using the proposed Gaussian process regressor could improve classification performances for all models. However, adding too many GP-generated, artificial samples leads to stagnation or even decline of F1-score. This can be explained by the nature of our artificial data. They represent the general look of broken sequences very well but do not vary significantly amongst each other. Thus, adding too many of those very similar sequences does not add more information and can lead to even decreasing results. Detailed performance measures for LSTMFCN in Table 3 emphasize that adding too many synthetic, GP-generated sequences misleads the model. We see this in decreased accuracy, precision, and F1-score while recall increases.

## Conclusions

For seamless workflow in medical operation as well as cost reduction, early detection of broken or soon to brake MRI hardware is key. Thus, we employed sequential image features which carry information about the coil’s condition. We trained and tested three models, LSTM, FCN, and LSTMFCN. Furthermore, we generated representative, synthetic KPIs of broken coils using a Gaussian process regressor and thus, decreased the dominant data imbalance. This improved the F1-score of all our models. The overall best F1-score was found by adding 40 artificial samples using the LSTMFCN. We could improve the results compared to using the original data set without artificial data from F1-score of 87.45 to 92.30%. However, adding synthetic data using the GP regressor did not result in the expected push of classification performance for all amounts of added, synthetic data in our experiments. Using too many artificial sequences would lead to overfitting towards the synthetic data. For each problem statement, this sweet spot needs to be found experimentally. We finally could prove the power of combining LSTM, FCN, and data augmentation and solve the classification problem, successfully.

In practice, our proposed pipeline can be employed for MRI coil failure detection at the earliest possible state. Thus, the trained model continuously predicts the state of coils being normal or broken. If the model is implemented directly at the imaging device, real-time prediction and failure detection are possible before a patient was scanned. If the model is implemented in a different environment, data transfer times delay the prediction result. This still improves reaction times and coil exchange times.

In future work, more data should be incorporated for training and validated by expert knowledge. Furthermore, our KPIs are measured per coil element, whereas the label only applies on the entire coil which can contain broken and normal coil elements at the same time. As not all coil elements are used in every measurement, data can contain KPIs hinting to normal condition of the coil even if it is actually broken. Thus, further consideration should cover KPIs measured mirroring all coil elements. Moreover, the generation of synthetic data shall further be investigated. In this work, we only covered a basic approach of Gaussian process regressor. In future research, the Gaussian process regressor concept could be further enhanced by, e.g. shapelets and applying ideas of Unsupervised Feature Learning from Time Series (USLM) [19].

## References

[1] Karim F, Majumdar S, Darabi H, Chen S (2017). Lstm fully convolutional networks for time series classification. IEEE access.

[2] Duda RO, Hart PE, Stork DG (2012) Pattern classification. Wiley

[3] Bishop CM (2006) Pattern recognition and machine learning. Springer

[4] Cui Z, Chen W, Chen Y (2016) Multi-scale convolutional neural networks for time series classification. arXiv:1603.06995 10.1016/j.neunet.2021.01.001 33485098

[5] Hochreiter S, Schmidhuber J (1997). Long short-term memory. Neural Comput.

[6] Sundermeyer M, Schlüter R, Ney H: Lstm neural networks for language modeling.. In: Thirteenth annual conference of the international speech communication association, 2012

[7] Ordóñez FJ, Roggen D (2016). Deep convolutional and lstm recurrent neural networks for multimodal wearable activity recognition. Sensors.

[8] Wang Z, Yan W, Oates T: Time series classification from scratch with deep neural networks: A strong baseline.. In: 2017 international joint conference on neural networks (IJCNN). IEEE, 2017, pp 1578–1585

[9] VanDyk DA, Meng X-L (2001). The art of data augmentation. J Comput Graph Stat.

[10] Williams DP, Myers V, Silvious MS (2009). Mine classification with imbalanced data. IEEE Geosci Remote Sens Lett.

[11] Davari A, Özkan HC, Maier A, Riess C: Fast sample generation with variational bayesian for limited data hyperspectral image classification.. In: IGARSS 2018-2018 IEEE International Geoscience and Remote Sensing Symposium. IEEE, 2018, pp 6159–6162

[12] Lorch B, Vaillant G, Baumgartner C, Bai W, Rueckert D, Maier A (2017) Automated detection of motion artefacts in mr imaging using decision forests. Journal of medical engineering 10.1155/2017/4501647 PMC5485319 28695126

[13] Jain B, Kuhnert N, deOliveira A, Maier A: Image-based detection of mri hardware failures.. In: Bildverarbeitung für die Medizin 2019. Springer, 2019, pp 206–211

[14] Kuhnert N, Pflüger L, Maier A: Prediction of mri hardware failures based on image features using ensemble learning.. In: Bildverarbeitung für die Medizin 2020. Springer, 2020, pp 137–142

[15] Chigurupati A, Thibaux R, Lassar N: Predicting hardware failure using machine learning.. In: 2016 Annual Reliability and Maintainability Symposium (RAMS). IEEE, 2016, pp 1–6

[16] Kuhnert N, Pflüger L, Maier A: Prediction of mri hardware failures based on image features using time series classification.. In: Bildverarbeitung für die Medizin 2020. Springer, 2020, pp 131–136

[17] Lipton ZC, Kale DC, Elkan C, Wetzel R (2015) Learning to diagnose with lstm recurrent neural networks. arXiv:1511.03677

[18] Williams CKI, Rasmussen CE (2006). Gaussian processes for machine learning.

[19] Zhang Q, Wu J, Yang H, Tian Y, Zhang C Unsupervised feature learning from time series, 2016, 2322–2328

